# Forced Fusion in Multisensory Heading Estimation

**DOI:** 10.1371/journal.pone.0127104

**Published:** 2015-05-04

**Authors:** Ksander N. de Winkel, Mikhail Katliar, Heinrich H. Bülthoff

**Affiliations:** 1 Department of Human Perception, Cognition, and Action, Max Planck Institute for Biological Cybernetics, Spemanstrasse 38, 72076 Tübingen, Germany; 2 Department of Brain and Cognitive Engineering, Korea University, Anam-dong, Seongbuk-gu, Seoul 136-713, Korea; Durham University, UNITED KINGDOM

## Abstract

It has been shown that the Central Nervous System (CNS) integrates visual and inertial information in heading estimation for congruent multisensory stimuli and stimuli with small discrepancies. Multisensory information should, however, only be integrated when the cues are redundant. Here, we investigated how the CNS constructs an estimate of heading for combinations of visual and inertial heading stimuli with a wide range of discrepancies. Participants were presented with 2s visual-only and inertial-only motion stimuli, and combinations thereof. Discrepancies between visual and inertial heading ranging between 0-90° were introduced for the combined stimuli. In the unisensory conditions, it was found that visual heading was generally biased towards the fore-aft axis, while inertial heading was biased away from the fore-aft axis. For multisensory stimuli, it was found that five out of nine participants integrated visual and inertial heading information regardless of the size of the discrepancy; for one participant, the data were best described by a model that explicitly performs causal inference. For the remaining three participants the evidence could not readily distinguish between these models. The finding that multisensory information is integrated is in line with earlier findings, but the finding that even large discrepancies are generally disregarded is surprising. Possibly, people are insensitive to discrepancies in visual-inertial heading angle because such discrepancies are only encountered in artificial environments, making a neural mechanism to account for them otiose. An alternative explanation is that detection of a discrepancy may depend on stimulus duration, where sensitivity to detect discrepancies differs between people.

## Introduction

Estimation of the direction of horizontal linear self-motion, or heading, is essential to human locomotion. Heading can be estimated from both visual and inertial information. Visually, heading is specified in the optic flow pattern: to an observer traveling along a straight path, heading is specified by the point from which the optic flow radially expands [1]. Other information on heading is provided by our inertial sensors: the otoliths of the vestibular system [2], and a variety of somatosensory sensors distributed throughout the body that either directly or indirectly respond to the magnitude and direction of accelerations of the body [3, 4].

There is ample evidence that the Central Nervous System (CNS) combines heading information provided by the different sensory channels. Neurophysiological research in macaque monkeys has shown that visual and vestibular afferents may converge in the CNS on several locations. The first evidence was provided by [5], who found that the vestibular nuclei were also responsive to optokinetic patterns (but see: [6]). The cortical area currently considered the most likely site of convergence of visual-vestibular heading information is the dorsal medial superior temporal area (MSTd) [6, 7], as neurons at this site have been shown to respond to optic flow patterns suggesting self-motion [8–14] as well as to translation in absence of visual information [15–18]. Moreover, this area could be responsible for the formation of an integrated percept out of the multisensory cues [19, 20]. The observed neural interconnectivity between the visual and vestibular channels is also reflected by behavioral data, as it has been shown that visual-inertial heading estimation is consistent with statistically optimal multisensory cue integration (e.g., [21–24]) in both non-human primates [25, 26], as well as in human subjects [25–28] for congruent multisensory stimuli and stimuli with small discrepancies [29].

Multisensory information should, however, only be integrated when cues are redundant [30, 31]: the cues must share a common cause. Because motion registered by the visual system can be caused both by self- and object motion, it is not trivial that integration occurs. Indeed, in one recent study it was found that human observers relied completely on the inertial component of multisensory motion stimuli for heading estimation, despite the fact that inertial heading estimation was less precise than visual heading estimation [32]. In this study, the visual stimulus was presented without disparity information, which may have led to a violation of the redundancy condition, as it has been found that the presence of disparity information increases the incidence of statistically optimal integration [28].

The fact that integration of visual- and inertial heading cues occurs only when particular conditions are met implies that the CNS assesses causality for visual and inertial heading cues. A model that explicitly performs causal inference was proposed by [33], to explain auditory-visual spatial localization. This model, designated Causal Inference (CI) model by the authors, is based upon the assumption that the CNS attempts to minimize a particular cost-function: the mean squared error between the final estimate of some environmental property and its true value. The result is that the final estimate is a weighted average of the estimates derived for the two possible causal structures (i.e., common cause vs. independent causes). Because of this, the strategy has been referred to as *Model Averaging* [34]. Plausible alternatives to this strategy are a simultaneous minimization of the error in estimated causal structure and in the spatial estimate, or to choose between causal structures based on the probability of either structure occuring [34, 35]. These two alternatives are known as *Model Selection* and *Probability Matching*, respectively.

In the present study, we developed analogues of the three alternative CI models discussed in the previous paragraph, tailored to account for the circular nature of heading information (e.g., [36, 37]). The tenability of each model was assessed by comparing the agreement between the predictions made by the models to heading estimates from human participants in response to visual-inertial motion stimuli with discrepancies of various sizes.

## Materials and Methods

### Ethics Statement

The experiment was conducted in accordance with the Declaration of Helsinki. All participants gave their written informed consent prior to participation. The experimental protocol and consent forms were approved by the ethical commision of the medical faculty of the Eberhard-Karls University in Tübingen, Germany.

### Apparatus

The experiment was performed using the Max-Planck Institute CyberMotion Simulator. This is an anthropomorphic robot arm fitted with an additional cabin (KUKA Roboter GmbH, Augsburg, Germany, model Robocoaster; [38]). The cabin has a double-curved projection screen with dual projectors, with a field of view of approximately 140° horizontal by 70° vertical. The dual projection system was used in conjunction with stereo glasses (Infitec®GmbH, Ulm, Germany, model INFITEC®Premium Glasses) to generate stereoscopic visual stimuli. The area of overlap between projectors used for stereo projection is approximately 90° by 70°. Participants were seated within the simulator cabin and secured by a five-point safety harness. Participants wore ear-enclosing headphones that dimmed simulator noise. An integrated microphone allowed permanent communication with the experimenter. Responses were provided via a custom-made pointer device. The pointer device consisted of a stainless steel rod of about 20cm that was connected to a potentiometer in its middle. One end of the rod was covered by 5cm of black heat shrink tubing. The device was placed on an aluminum frame right above the participant’s laps. Participants were instructed to hold the rod by the covered end with their right hand; the other end was to be interpreted as the arrow’s head. The rod could be rotated in the horizontal plane, did not have any discontinuities, and provided a < 0.1° resolution. It was visible to the participants during the response phase of all stimuli.

### Task & Stimuli

Stimuli consisted of presentation of visual and/or inertial horizontal linear motions with different headings. For both modalities the motion profile was a raised cosine bell in velocity, specified as

$$\begin{array}{c} v=\frac{v_{max}}{2}\left(1-cos\omega t\right) \end{array}$$ (1)

With *ω* = 2*πf*, *f* = 0.5Hz, and *v*_*max*_ = 0.3m/s (*a*_*max*_ ≈ 0.5m/s^2^, *x*_*max*_ = 0.3m).

**Fig 1:**
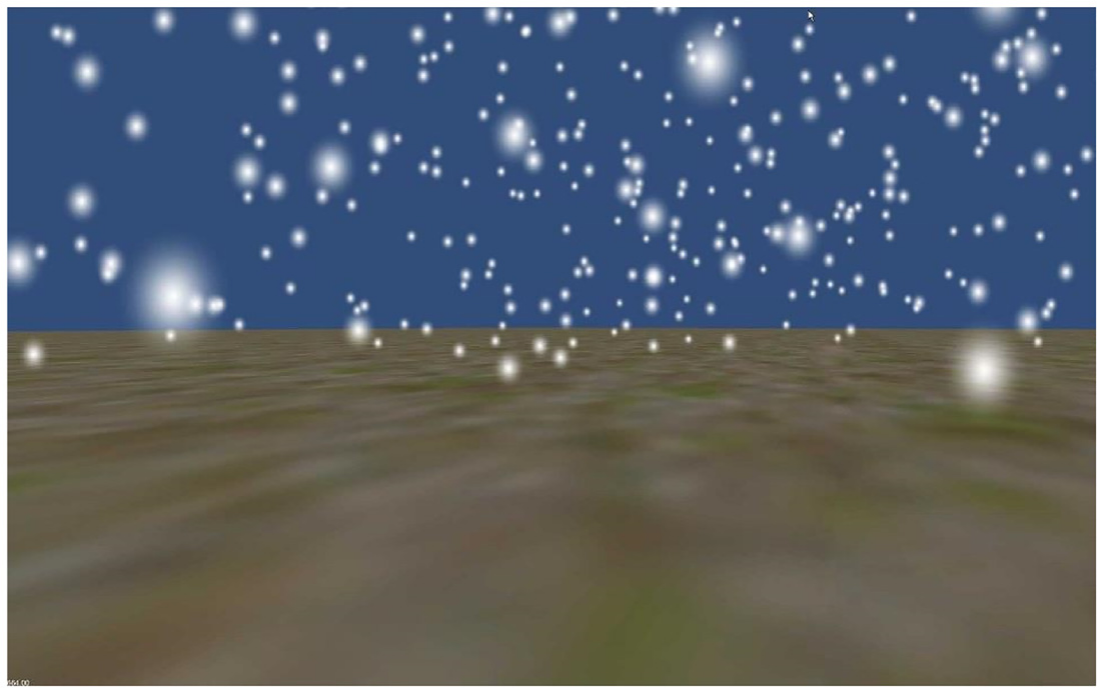
Visual scene. To provide disparity information, the visual stimuli were presented stereoscopically using a dual-projector setup. The figure only shows the image presented by one projector.

The visual motions consisted of movement through a three dimensional environment with a ground plane and limited-lifetime particles (Fig 1).

The ground plane was blurred to make it virtually impossible for participants to determine the heading of visual motion by tracking a particular point. The particles appeared at random times and positions in a large spherical volume around the main camera. Each particle disappeared after a 0.3s lifetime. Visual stimuli were generated using the Unity game engine (Unity Technologies, San Francisco, United States, version Unity 4.2.2f1), and presented stereoscopically. The particularities of the visual environment were determined in pilot experiments, and chosen such that performance in terms of response variability in the visual- and inertial-only conditions was approximately equal.

**Fig 2:**
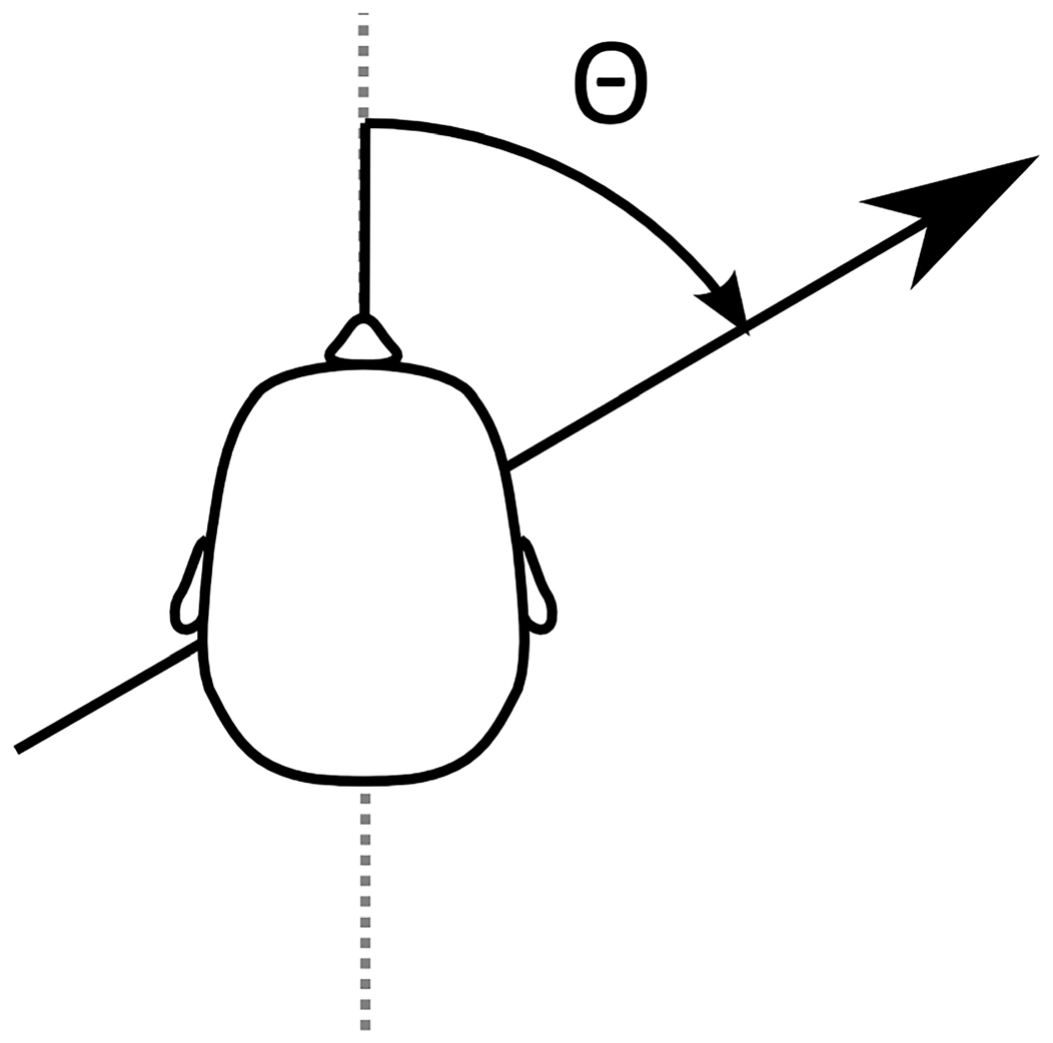
Operationalization of heading angle. The participant was oriented at angle *θ* relative to the simulator motion path (represented by black arrow). For straight ahead motion, the participant’s naso-occipital axis (dashed gray line) would be aligned with the simulator motion path.

Inertial motions were generated by moving the simulator cabin either forward or backward over a distance of 0.3m along an imaginary horizontal line. Different headings were achieved by varying the cabin’s yaw angle relative to this line (Fig 2). The direction in which the simulator was moved (forward or backward) was chosen separately for each stimulus, in such a way that the duration of reorientation between stimuli was minimal (See Supporting Information S1 Video and S2 Video for animations of forward and backward motions, respectively).

There were three different experimental conditions: a visual-only condition, an inertial-only condition, and a ‘combined’ condition.

In both the visual- and inertial-only conditions, stimuli were presented with headings ranging between −180° (−*π* rad) and 180° (*π* rad), where 0 heading corresponds to straight-ahead motion, and positive values are orientated away from 0 in the clockwise direction. Per condition 180 stimuli were presented, with headings in the aforementioned range distributed in evenly spaced steps.

The stimuli presented in the combined condition were 180 discrepant combinations of visual and inertial motions, randomly generated for each participant. Visual motions with headings evenly spaced between −180° and 180° (every 2°) were coupled with inertial motions with headings that were the sum of the visual heading and a deviation drawn from a normal distribution with a mean of 0 and a variance of 50°, resulting in an effective maximum discrepancy of $90^{{}^{\circ}}\;(\frac{1}{2}\pi \;\text{rad})$. This range includes discrepancies that are below as well as above the threshold for detection of a discrepancy, given that 50% correct detection of a discrepancy between a visual motion stimulus with 0° heading and an inertial motion stimulus has been estimated to occur at ±14° (0.24, rad) [39].

The task participants had to perform was to use the pointer device to indicate the heading of self-motion after playback of each stimulus. Because the visual-only stimuli did not induce a sensation of actual self-motion, participants received the additional instruction to indicate the heading of self-motion that was suggested by the visual stimulus for visual-only trials.

### Procedure

Prior to the experiment, participants were given a general explanation of the experimental goals and procedures, and given the opportunity to ask additional questions. They were instructed to keep their gaze focused on the central part of the projection screen, and to give their heading judgments as quickly and accurately as possible after a motion was completed. A fixation cross was not implemented. It has been shown that a fixation cross does not affect inertial heading estimation [40, 41]. Moreover, on a theoretical basis, a fixation cross could be used to track a particular element of the optic array relative to the fixation cross, changing the nature of the task. After giving their informed consent, participants completed a series of 10 random stimuli in order to get familiar with the setup and the task.

After familiarization, the actual experiment started. Stimuli of the visual-only, inertial-only and combined conditions were presented intermixed, in a random order that was defined at onset of the experiment. Playback of stimuli with visual motion was preceded by a high pitched beep (0.5s, 1760Hz); stimuli without visual motion were preceded by a low-pitched beep (0.5s, 440Hz). Participants were instructed to keep their eyes closed for stimuli without a visual component (i.e., after a low-pitched beep). Participants were monitored during the training phase of the experiment and reminded of these instructions if they did not follow them.

At the onset of each stimulus, the cabin was realigned to the stimulus’ heading angle, or, in case the stimulus was visual-only, the heading angle of the first next stimulus with an inertial motion component. The duration of this reorientation depended on the difference between the simulator’s current heading angle and the heading angle of the stimulus, and the direction of linear motion of the simulator cabin. Cabin motion could be forward or backward along the simulator motion path, which was fixed in space. Because the order of stimuli, their nature (i.e., visual-only, inertial-only, or combined), and the direction of the linear motion were randomized, the duration of reorientation could not be used as a cue to estimate the heading of the next stimulus.

The maximum angular acceleration and velocity for reorientation were 4.98°/s^2^ (0.087 rad/s^2^) and 10.03°/s (0.175 rad/s), respectively. A 2s break was implemented after the reorientation phase to allow any after-effects to fade.

The total number of 540 stimuli were presented in four blocks of approximately 135 trials, where a trial constitutes a stimulus-response cycle. Each block took about 30 minutes to complete. Including instruction and mandatory breaks between blocks, the experiment took between 4–4.5 hours to complete.

### Participants

Twelve participants (5F) were recruited to take part in the experiment. The participants all reported minimal susceptibility to motion sickness and claustrophobia, and no history of any intestinal, neurological, or vestibular illnesses. Due to safety regulations, participation was only allowed for people between 18–65 years old, weighing up to 90kg, and measuring at most 1.95m. Data of three participants had to be excluded from the analysis due to either an apparent misinterpretation of the instructions (2 cases: responses were limited to the cardinal axes), or due to an inability to discern stimulus headings (zero correlation between stimulus heading and response heading). Because of the nature of the responses provided by these participants, we could not characterize their unisensory heading estimation, and consequently could not assess the nature of multisensory processing.

### Data Analysis

Analysis of the data was completed in two steps. In the first step, models of unisensory heading estimation were fitted to the data obtained in the unisensory conditions. In the second step of the analysis, the tenability of a number of models of multisensory heading was assessed, which relied on the parameters obtained in the first step. The analyses were performed separately for each participant.

**Unisensory Models** The model of unisensory heading estimation was:

$$\begin{array}{c} R=x∼𝓜(\mu ,\kappa ) \end{array}$$ (2)

with

$$\begin{array}{c} \mu =\beta_{0}+\mathrm{atan}\left(\beta_{1}\sin\theta ,\cos\theta \right) \end{array}$$ (2a)

$$\begin{array}{c} \kappa =\gamma_{0}-\gamma_{1}\left|\sin2\theta \right| \end{array}$$ (2b)

Here *R* represents the response, which is assumed to be equal to the output of the sensory system, signal *x*. ℳ(*μ*, *κ*) represents a von Mises distributed random variable with mean *μ* and dispersion parameter *κ*. The von Mises distribution is a circular analogue of the normal distribution [42]. The mean *μ* is a function of the heading angle *θ* of the stimulus (in radians). The mean is composed of a parameter that reflects any constant offset in responses *β*_0_ and the four-quadrant inverse tangent of *y* = *β*_1_ sin *θ* and *x* = cos *θ*. Parameter *β*_0_ was assumed to be zero, but was included to allow for any deviations; parameter *β*_1_ affects the size of bias. To illustrate: a value of *β*_1_ = 1 indicates that there is no heading dependent bias; values of *β*_1_ < 1 indicate bias toward the fore-aft axis; and values of *β*_1_ > 1 indicate bias away from the fore-aft axis. The dispersion parameter *κ* is an analogue of the reliability $\frac{1}{\sigma^{2}}$ of a Normal distribution. It was expressed as the sum of a baseline level *γ*_0_ and a part that varies periodically with heading angle, with parameter *γ*_1_. The periodical nature of the bias and the dispersion in the model mimics experimental observations for patterns of bias and dispersion of both visual and vestibular heading estimation [26, 41, 43]. We assume that the CNS does not have any knowledge of these biases, as known biases can be compensated for. It should be noted that this model serves to characterize unisensory heading estimates, but does not reflect any assumptions on underlying generative mechanisms. For both unisensory conditions, there were four free parameters: *β*_0_, *β*_1_, *γ*_0_, and *γ*_1_. We assume that the likelihood of the parameters has a single strong peak, and consequently determine the optimal value for each of the parameters by maximum-likelihood estimation, using the MATLAB fminsearch routine (The MathWorks Inc., Natick, United States, version 2013b). To assess the goodness of fit, we calculated the Generalized Coefficient of Determination, R^2^. This measure compares the model likelihood to the likelihood of a null-model, which assumes zero bias *μ* = *θ*, and *κ* as a constant [44]. This measure ranges between zero and one, corresponding to a lack of improvement and strong improvement compared to the null model, respectively.

**Fig 3:**
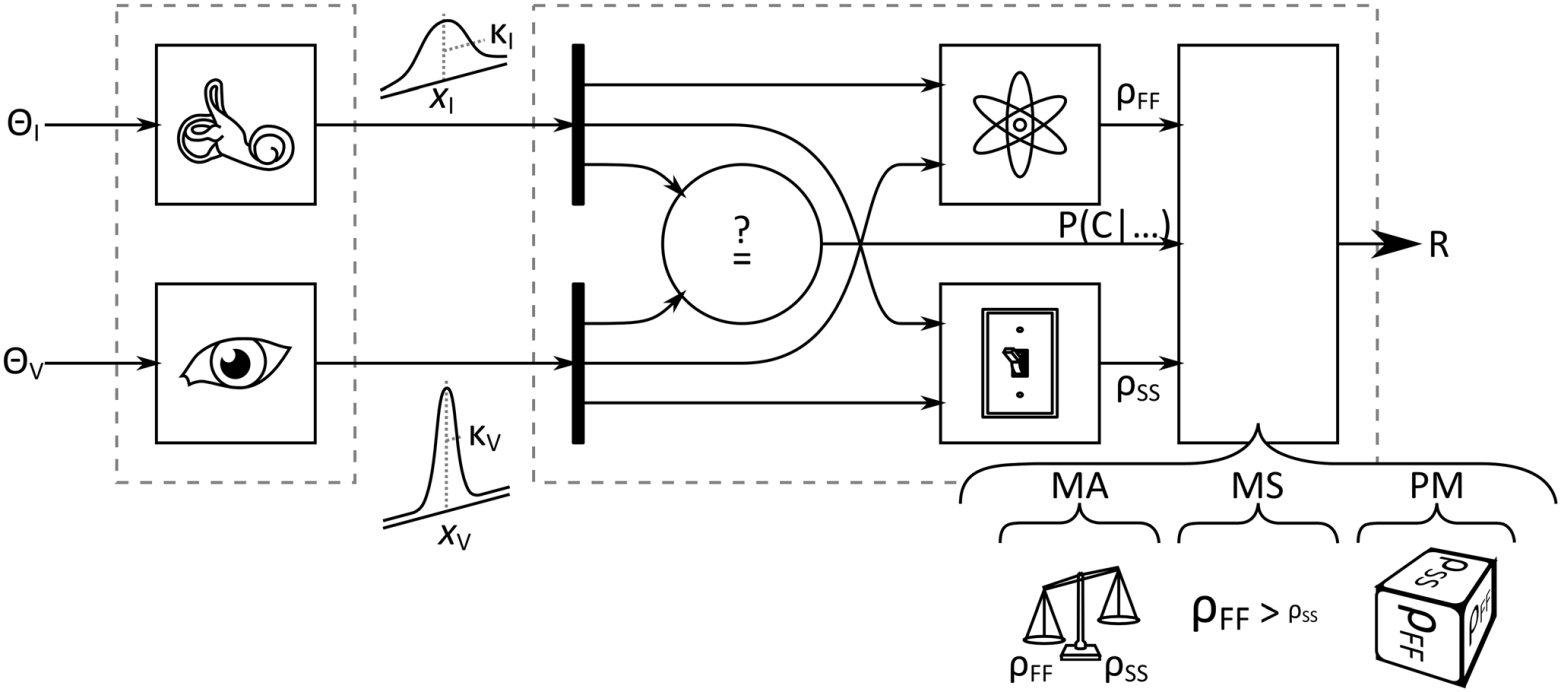
Schematic of the models. Sensory neurons are excited by stimuli *θ*_*V*_ and *θ*_*I*_, and pass representations of the stimuli on to the CNS. These internal representations carry average estimates *x*_*V*_ and *x*_*I*_, as well as information about their dispersions, *κ*_*V*_ and *κ*_*I*_. The probability that the internal representations share a common cause P(C | …) is assessed; a fused estimate *ρ*_*FF*_ is generated; and an individual representation *ρ*_*SS*_ is chosen. A final estimate *R* is generated by processing intermediate estimates *ρ*_*FF*_ and *ρ*_*SS*_ according to a particular strategy. These strategies take the estimated probability of a common cause into account.

**Multisensory Models** In the second step of the analysis, the fit of several alternative models of multisensory heading estimation was compared in order to assess how to best explain the observed multisensory heading estimates. The analysis is largely analogous to the approach taken by [34], adapted for circular stimuli. The models come from a ‘spectrum’ of strategies that reflect how the CNS could estimate heading when visual and inertial information is simultaneously available. A schematic for the models is provided in Fig 3.

On one end of the spectrum of models, the CNS fuses the signals provided by the visual and inertial system in a statistically optimal fashion, regardless of any discrepancy between them. By ‘statistically optimal’ we mean that the expected value of a certain cost function *c* of heading estimation error *R*−*S* is minimized given the a-posteriori belief about the heading *S*:

$$\begin{array}{c} R\left(x_{V},x_{I},\kappa_{V},\kappa_{I}\right)=\underset{R}{\mathrm{argmin}}\int_{-\pi }^{\pi }P\left(S\;|\;x_{V},x_{I},\kappa_{V},\kappa_{I}\right)c\left(R-S\right)dS \end{array}$$ (3)

It can be shown that if a) *c* is an even function and b) P(*S* | *x*_*V*_, *x*_*I*_, *κ*_*V*_, *κ*_*I*_) is unimodal and symmetric around its point of maximum *μ*, then the expression above is minimized if *R* = *μ*. The posterior is given by

$$\begin{array}{c} P\left(S\;|\;x_{V},x_{I},\kappa_{V},\kappa_{I}\right)\propto P\left(x_{V},x_{I}\;|\;S,\kappa_{V},\kappa_{I}\right)P\left(S\;|\;\kappa_{V},\kappa_{I}\right) \end{array}$$ (4)

As explained in the previous section for the unisensory case, we assume that the CNS treats visual and inertial representations of heading as veridical: it does not have any knowledge of sensory bias, and it is also unaware of the relation between heading angle and perceptual noise. Therefore, we assume that the belief about *S* does not depend on *κ*_*V*_, *κ*_*I*_: P(*S* | *κ*_*V*_, *κ*_*I*_) = P(*S*). Stated differently, this implies that *κ*_*V*_, *κ*_*I*_ do not provide additional information about *S*.

The dispersions *κ*_*V*_ and *κ*_*I*_ are treated as knowns, as it has been shown that the dispersion is present in the activity of populations of neurons (e.g., [45]). We calculated their respective sizes using the unisensory models, with values for parameters *β*_0_, *β*_1_, *γ*_0_, and *γ*_1_ estimated from the unisensory data. Given that *x*_*V*_ and *x*_*I*_ are independent, the posterior becomes a product of two von Mises distributions, which is itself von Mises distributed with parameters *μ*_*VI*_, *κ*_*VI*_:

$$\begin{array}{c} P\left(S\;|\;x_{V},x_{I},\kappa_{V},\kappa_{I}\right)\propto f_{VM}\left(x_{V};S,\kappa_{V}\right)f_{VM}\left(x_{I};S,\kappa_{I}\right)\propto f_{VM}\left(S;\mu_{VI},\kappa_{VI}\right) \end{array}$$ (5)

where *f*_*VM*_(⋅ | *μ*, *κ*) represents the von Mises probability density with mean parameter *μ* and dispersion parameter *κ*, and *x*_*V*_ and *x*_*I*_ are determined as in the unisensory models. The resulting distribution satisfies the conditions of being unimodal and symmetric around its mean *μ*_*VI*_. Therefore we take *μ*_*VI*_ as an estimator of *S*:

$$\begin{array}{c} R=\mu_{VI}=\mathrm{Arg}\left(\kappa_{V}e^{ix_{V}}+\kappa_{I}e^{ix_{I}}\right) \end{array}$$ (6)

This strategy will be henceforth referred to as Forced Fusion (FF). It is equivalent to Bayesian Integration schemes using a uniform prior, known as Maximum-Likelihood Integration, but uses circular distributions (e.g., [21–24]). A notable difference of the present implementation compared to models using Gaussian distributions is that the combined estimate can be less certain than the constituent estimates. For a detailed account on cue combination for circular data see [36].

On the other end of the spectrum of models, the CNS does not fuse visual and inertial signals at all, but relies on either source of information to construct response *R*. In the present experiment, participants were instructed to report the perceived heading of self-motion, thus possibly ignoring the visual stimulus. However, since previous work on cue combination in self-motion perception has shown that people may alternate between sources of information regardless of their instructions [32, 46], we allowed the source of the heading estimate to vary on a trial-to-trial basis. This strategy will be referred to as the Switching Strategy (SS; [46]), and can be thought of as the CNS flipping a biased coin to decide between the visual signal *x*_*V*_ and inertial signal *x*_*I*_:

$$\begin{array}{c} R=\{\begin{array}{cc} x_{V}, & \text{with}\;\text{probability}\;\xi \\ x_{I}, & \text{with}\;\text{probability}\;1-\xi \end{array} \end{array}$$ (7)

The probability of choosing a particular source is likely to depend on the task or instructions given to a participant. Consequently, we assumed that this parameter was constant throughout the experiment. Because its value was unknown and may differ between participants, probability *ξ* was treated as a free parameter.

In between the FF and SS models as extreme cases come Causal Inference (CI) models [33, 34]. In CI models, an assessment of the likelihood of alternative causal structures (i.e., a common cause or independent causes for *x*_*V*_ and *x*_*I*_) affects how the final estimate is constructed. There are myriad ways for the CNS to incorporate such an assessment in the construction of a final estimate. Plausible alternatives are *Model Averaging* (MA), *Model Selection* (MS), and *Probability Matching* (PM) [34, 35]. MA states that the final estimate is a weighted average of intermediate estimates *ρ*_*FF*_ and *ρ*_*SS*_ according to the FF and SS strategies, respectively. The weights are the respective probabilities of a common cause and independent causes. Because of the circular nature of heading estimates, we cannot simply take a weighted sum of the intermediate estimates. A weighted average of the intermediate estimates causes erroneous results at the extremes of possible headings (i.e. −*π* and *π*): consider the case where FF would yield an estimate of $-\frac{3}{4}\pi$ and SS an estimate of $\frac{3}{4}\pi$. The arithmetic mean would be zero, while intuitively, the mean should be *π* (or −*π*). Therefore, we represent the intermediate heading estimates as complex numbers, take a weighted sum, and revert the resulting complex representation *z* back to a heading angle:

$$\begin{array}{c} R_{\mathrm{MA}}=\mathrm{Arg}\;z_{MA} \end{array}$$ (8)

where

$$\begin{array}{c} z_{\mathrm{MA}}=P\left(C\;|\;x_{V},x_{I},\kappa_{V},\kappa_{I}\right)e^{i\rho_{FF}}+P\left(\bar{C}\;|\;x_{V},x_{I},\kappa_{V},\kappa_{I}\right)e^{i\rho_{SS}} \end{array}$$ (8a)

and

$$\begin{array}{c} \rho_{FF}=\mathrm{Arg}\left(\kappa_{V}e^{ix_{V}}+\kappa_{I}e^{ix_{I}}\right) \end{array}$$ (8b)

$$\begin{array}{c} \rho_{SS}=\{\begin{array}{cc} x_{V}, & \text{with}\;\text{probability}\;\xi \\ x_{I}, & \text{with}\;\text{probability}\;1-\xi \end{array} \end{array}$$ (8c)

Here P(C | *x*_*V*_, *x*_*I*_, *κ*_*V*_, *κ*_*I*_) represents the probability of a common cause given signals *x*_*V*_ and *x*_*I*_ and their respective dispersions *κ*_*V*_ and *κ*_*I*_, and $\text{P}(\bar{\text{C}}\;|\;x_{V},x_{I},\kappa_{V},\kappa_{I})$ represents the probability of independent causes. A discussion on the properties and calculation of the probability of a common cause given a-priori beliefs and sensory information P(C | *x*_*V*_, *x*_*I*_, *κ*_*V*_, *κ*_*I*_) is provided in the section Probability of causal structures.

For MS, the final estimate is either the result of FF or SS, depending on which causal structure is more likely: a common cause or independent causes, respectively.

$$\begin{array}{c} R_{\mathrm{MS}}=\{\begin{array}{cc} \rho_{FF}, & \text{if}\;P\left(C\;|\;x_{V},x_{I},\kappa_{V},\kappa_{I}\right)\geq \frac{1}{2}\\ \rho_{SS}, & \text{if}\;P\left(C\;|\;x_{V},x_{I},\kappa_{V},\kappa_{I}\right)<\frac{1}{2} \end{array} \end{array}$$ (9)

The PM decision strategy is very similar to MS, but the decision to base the final estimate on either SS or FF is made at random, where the probability of deciding in favor of SS is equal to the probability of a common cause.

$$\begin{array}{c} R_{\mathrm{PM}}=\{\begin{array}{cc} \rho_{FF}, & \text{with}\;\text{probability}\;P(C\;|\;x_{V},x_{I},\kappa_{V},\kappa_{I})\\ \rho_{SS}, & \text{with}\;\text{probability}\;1-P(C\;|\;x_{V},x_{I},\kappa_{V},\kappa_{I}) \end{array} \end{array}$$ (10)

**Probability of causal structures** There are two possible causal structures for two sensory signals: either the signals share a common cause, C, or they do not, $\bar{\text{C}}$. The probability of a common cause given a set of sensory signals P(C | *x*_*V*_, *x*_*I*_, *κ*_*V*_, *κ*_*I*_) can be found using Bayes’ rule:

$$\begin{array}{c} P\left(C\;|\;x_{V},x_{I},\kappa_{V},\kappa_{I}\right)\propto P\left(x_{V},x_{I}\;|\;C,\kappa_{V},\kappa_{I}\right)P\left(C\right) \end{array}$$ (11)

Here P(C) is the prior probability of a common cause, which we assume to be independent of *κ*_*V*_, *κ*_*I*_. It should be noted that parameter P(C) only represents an observer’s a-priori beliefs about whether or not cues will have a common cause (i.e., in the absence of any evidence). The a-posteriori probability of visual and inertial signals having a common cause is proportional to the a-priori beliefs on the one hand and the particular values and dispersions of the internal signals on the other. A value of parameter P(C) *close* to 1 does not imply that the model behaves almost identically to the Forced Fusion model: when this parameter is close, but not equal to one, this allows the model to attribute high common cause probabilities to multisensory stimuli with small discrepancies, and near zero common cause probabilities to multisensory stimuli with larger discrepancies, and allows for a different decision strategy when the sensory signals provide strong evidence that the stimuli are incongruent (i.e., P(*x*_*V*_, *x*_*I*_ | C, *κ*_*V*_, *κ*_*I*_) is close to 0).

The probability of a set of sensory signals *x*_*V*_ and *x*_*I*_ given a common cause can be found by calculating the integral of the probability of the signals over all possible values for *S*:

$$\begin{array}{c} P\left(x_{V},x_{I}\;|\;C,\kappa_{V},\kappa_{I}\right)=\int_{-\pi }^{\pi }P\left(x_{V},x_{I}\;|\;S,\kappa_{V},\kappa_{I}\right)P\left(S\right)dS \end{array}$$ (12)

Assuming a uniform prior (i.e., all heading angles are equally likely to occur) and independence of the visual and inertial channels, this becomes

$$\begin{array}{c} P\left(x_{V},x_{I}\;|\;C,\kappa_{V},\kappa_{I}\right)=\frac{1}{2\pi }\int_{-\pi }^{\pi }f_{VM}\left(x_{V}\;|\;S,\kappa_{V}\right)f_{VM}\left(x_{I}\;|\;S,\kappa_{I}\right)dS=\\ =\frac{1}{2\pi }\int_{-\pi }^{\pi }f_{VM}\left(S\;|\;\mu_{VI},\kappa_{VI}\right)\frac{2\pi I_{0}\left(\kappa_{VI}\right)}{2\pi I_{0}\left(\kappa_{V}\right)2\pi I_{0}\left(\kappa_{I}\right)}dS=\frac{I_{0}\left(\kappa_{VI}\right)}{4\pi^{2}I_{0}\left(\kappa_{V}\right)I_{0}\left(\kappa_{I}\right)} \end{array}$$ (13a)

$$\begin{array}{c} \kappa_{VI}=\;|\;\kappa_{V}e^{ix_{V}}+\kappa_{I}e^{ix_{I}}\;|\;=\;|\;\kappa_{V}+\kappa_{I}e^{i(x_{I}-x_{V})}| \end{array}$$ (13b)

Here *I*_0_(…) is the modified Bessel function of the first kind and order zero. *κ*_*V*_ and *κ*_*I*_ are treated as if they are independent of *S*, which reflects the assumption that any relation between the size of the dispersion and heading angle is unknown to the CNS (see: Unisensory Models). Put in words, the probability of a common cause given a set of sensory signals is equal to the total probability that any common cause *S* gave rise to both sensory signals. The less noisy the individual signals are, the more narrow the range of discrepancies that lead to a ‘common cause’ judgment will be.

Similarly, provided that the sensory channels are independent, the probability of sensory signals *x*_*V*_ and *x*_*I*_ given independent causes, $\text{P}(x_{V},x_{I}\;|\;\bar{\text{C}},\kappa_{V},\kappa_{I})$, is

$$\begin{array}{c} P\left(x_{V},x_{I}\;|\;\bar{C},\kappa_{V},\kappa_{I}\right)=\int_{-\pi }^{\pi }P\left(x_{V}\;|\;S_{V},\kappa_{V}\right)P\left(S_{V}\right)dS_{V}\int_{-\pi }^{\pi }P\left(x_{I}\;|\;S_{I},\kappa_{I}\right)P\left(S_{I}\right)dS_{I} \end{array}$$ (14)

With uniform priors, this becomes

$$\begin{array}{c} P\left(x_{V},x_{I}\;|\;\bar{C},\kappa_{V},\kappa_{I}\right)=\frac{1}{4\pi^{2}} \end{array}$$ (15)

The last expression means that assuming no prior information and incongruent stimuli, all combinations of sensory outputs *x*_*V*_ and *x*_*I*_ are equiprobable (i.e., a 2-dimensional uniform distribution for circular data).

**Fig 4:**
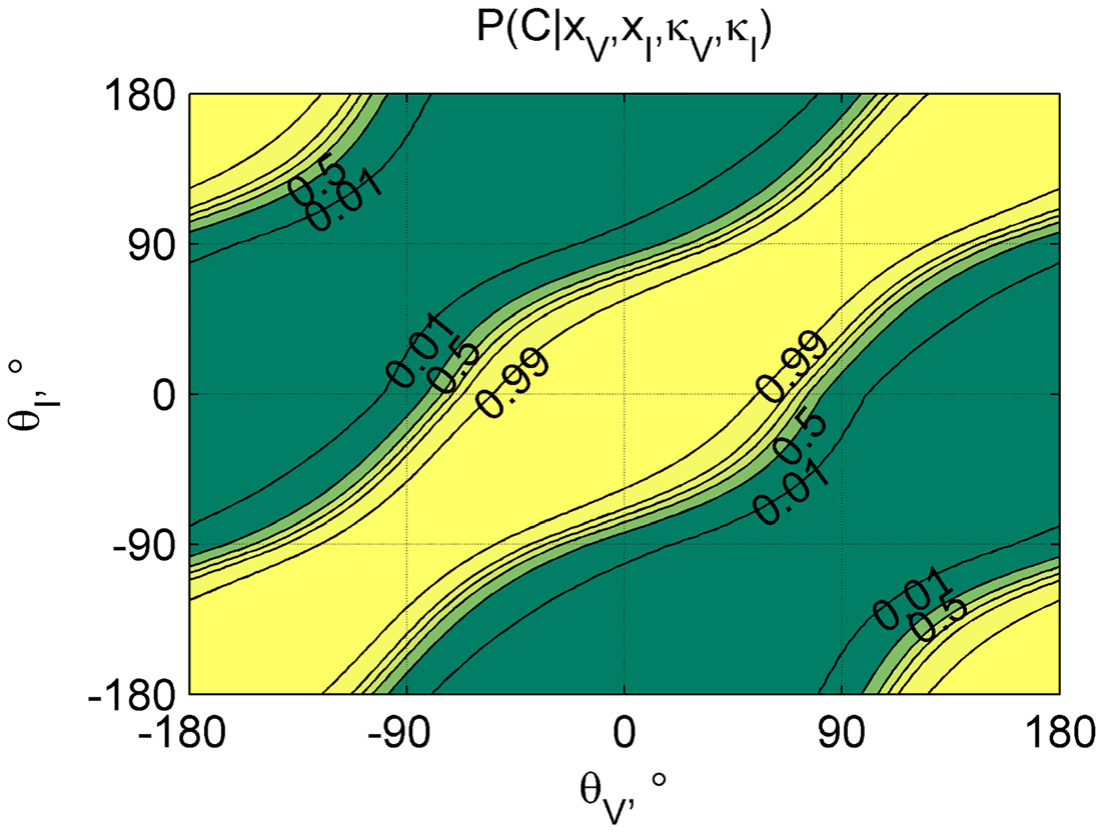
Probability of a common cause. The value of P(C | *x*_*V*_, *x*_*I*_, *κ*_*V*_, *κ*_*I*_) as a function of *θ*_*V*_, *θ*_*I*_; the visual and inertial heading angles, respectively. Values calculated using data of an example participant (7).

An illustration of how P(C | *x*_*V*_, *x*_*I*_, *κ*_*V*_, *κ*_*I*_) varies for different combinations of *θ*_*V*_, *θ*_*I*_, the visual and inertial heading angles, is provided in Fig 4. Note that for values for P(C) of 0 or 1, the surface becomes flat.

**Model Fitting & Selection** In the models of multisensory heading estimation, the prior probability of a common cause P(C) and reliance on the visual signal *ξ* for the SS-component were free parameters. Because the likelihoods of the CI models are intractable, we could not obtain estimates for these parameters analytically: the model likelihood is $P(R\;|\;\theta_{V},\theta_{I})=\int_{-\pi }^{\pi }\int_{-\pi }^{\pi }P(R\;|\;x_{V},x_{I})P(x_{V},x_{I}\;|\;\theta_{V},\theta_{I})dx_{V}dx_{I}$, where *P*(*x*_*V*_, *x*_*I*_ | *θ*_*V*_, *θ*_*I*_) = *f*_*VM*_(*x*_*V*_;*μ*_*V*_(*x*_*V*_), *κ*_*V*_(*x*_*V*_))*f*_*VM*_(*x*_*I*_; *μ*_*I*_(*x*_*I*_), *κ*_*I*_(*x*_*I*_)). *μ*_*V*_, *κ*_*V*_, *μ*_*I*_, *κ*_*I*_ are calculated from Eqs (2a) and (2b), and *P*(*R* | *x*_*V*_, *x*_*I*_) is calculated based on Eqs (7)–(15), depending on decision strategy. We could not compute the resulting integrals analytically. We therefore used the models specified above to generate large sets of simulated responses for given sets of parameters P(C) and *ξ*, and assessed the similarity of the simulations with the actual observations. Specifically, 1000 responses were simulated for each of the approximately 180 completed stimulus-pairs. For each stimulus pair, the likelihood of the actual response was derived from kernel density estimation using the simulated responses [47, 48]. The ‘circ_ksdensity’ kernel density estimation procedure for periodic data made for MATLAB was used to obtain density estimates [49]. The sum of the logarithm of the estimated probability densities for each of the responses forms the model’s log-likelihood. The optimal values for P(C) and *ξ* are those for which the likelihood attains its maximum value. Optimization was done using the MATLAB ‘patternsearch’ algorithm.

Because the fit of each model depends to some extent on the particular values of the simulated responses, the complete procedure was repeated 100 times for each model, using different random seeds. For 1000 simulated responses for each stimulus-pair, the observed variability in obtained log-likelihoods amounted to about 1.6%. For each model, the best fit obtained was chosen as the final estimate. The model with the smallest Bayesian Information Criterion score (BIC; [50]) was considered the best explanation of the observations. The BIC score is a measure of relative model quality based on the likelihood, and includes a penalty for the number of free parameters in the model. Simulations were done on a desktop computer with 3.60 GHz quad-core Intel Xeon processor and 16GB of RAM, and took about one week to complete.

We could not assess the goodness of fit of the multisensory models by means of the Generalized Coefficient of Determination R^2^, as was done to assess the fit of the unisensory models. Calculation of this statistic requires the specification of a null-model similar to the zero bias model used as comparison in the unisensory case. Since there is no single true heading angle for a discrepant combination of heading stimuli, such a null-model could not be defined for the multisensory case.

## Results

Data were analyzed on an individual level because the literature shows considerable differences between participants and studies; both in biases in unisensory heading estimation [41, 43, 51–54], as well as in multisensory processing [34]. For each participant, the data were analyzed in two steps. In the first step, data gathered in the unisensory conditions were used to estimate parameters of models of unisensory heading estimation. These models were used as components of the generative models of multisensory heading estimation in the second step of the analysis. To determine which model best described the data, the agreement of the simulations to the actual observations was assessed.

### Unisensory Conditions

**Fig 5:**
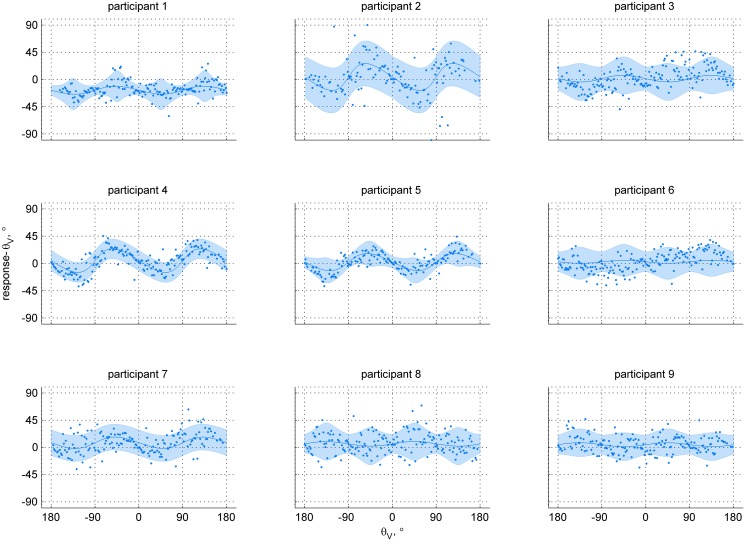
Individual data points and the fitted models for the visual-only condition. The panels show the data for each of the participants separately. The abscissa represents the stimulus heading angle; the ordinate represents the difference between the reported heading and the stimulus heading. The blue line is the model mean response *μ* minus stimulus heading for the range of stimuli; the shaded area represents the 95% CI. Each dot is a single data point.

**Fig 6:**
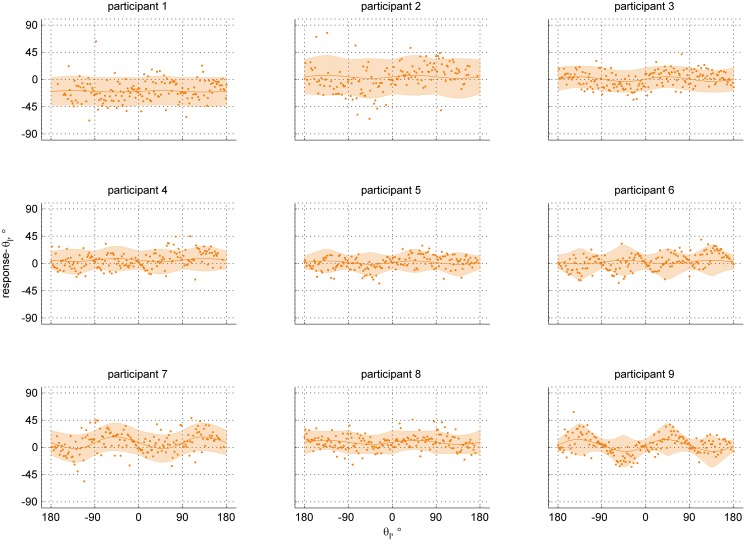
Individual data points and the fitted models for the inertial-only condition. The panels show the data for each of the participants separately. The abscissa represents the stimulus heading angle; the ordinate represents the difference between the reported heading and the stimulus heading. The orange line is the model mean response *μ* minus stimulus heading for the range of stimuli; the shaded area represents the 95% CI. Each dot is a single data point.

An overview of the unisensory data is presented in Figs 5 and 6, for the visual and inertial conditions respectively. The panels of each figure show the data and fitted unisensory models for each participant. The obtained R^2^ statistics ranged between 0.042 and 0.780, indicating small to large improvement over a model assuming zero bias and a constant kappa (Supporting Information S1 Table).

Data for the visual-only condition show bias towards the fore-aft axis for seven out of nine participants (as indicated by a *β*_1_ < 1, Supporting Information S2 Table). The maximum bias according to the fitted models ranges between 2.39° (0.04rad, participant 6) and 22.28° (0.39rad, participant 2), with a median value across participants of 6.59°. The remaining two participants show a slight bias away from the fore-aft axis. In the inertial-only condition, we found bias away from the fore-aft axis for six out of nine participants (as indicated by a *β*_1_ > 1, Supporting Information S2 Table). According to the fitted models, the maximum bias ranges between 1.12° (0.02rad, participant 1) and 9.80° (0.17rad, participant 9), with a median value across participants of 2.97°. The other three participants show a slight bias towards the fore-aft axis. The plateaus observed in the inertial-only condition for participants six and nine are thought to be a spurious result caused by a tendency to align the rod with the cardinal axes.

### Multisensory Condition

We assessed which of the alternative models provided the best explanation of the responses in the multisensory conditions by comparing the models’ BIC-scores. These scores are a measure of relative model quality (see: Model Fitting & Selection). BIC-scores for the alternative models of multisensory heading perception are presented in Table 1. The best fitting model was FF for five participants; for the remaining participants the data were best explained by the MA model. The maximum-likelihood estimates of the parameters P(C) and *ξ* for the participants for whom the causal inference model provided the best fit are presented in Table 2.

**Table 1:** Model BIC scores.

	BIC				
pp	FF	SS	MA	MS	PM
1	**123.93**	160.09	130.47	130.63	130.63
2	**196.32**	235.45	197.90	198.99	199.86
3	**134.21**	173.33	136.36	136.47	136.37
4	87.83	108.51	**82.83**	89.36	88.95
5	6.80	64.29	**5.97**	8.35	8.44
6	**95.58**	154.72	99.06	99.44	99.67
7	135.54	170.75	**133.70**	135.03	135.12
8	**98.16**	138.90	102.18	102.26	102.05
9	81.10	118.92	**79.20**	80.35	80.07

The abbreviation ‘pp’ stands for participant; boldfaced values correspond to the ‘best’ BIC-score. For an explanation of the models and their acronyms see methods section: Multisensory Models

**Table 2:** Causal Inference model parameter estimates.

	MA	
pp	P(C)	*ξ*
4	0.94933	0.00004
5	0.99998	1.00000
7	0.99885	0.00000
9	0.99938	0.00000

The abbreviation ‘pp’ stands for participant. For an explanation of the parameters P(C) and *ξ* see methods section: Multisensory Models

**Fig 7:**
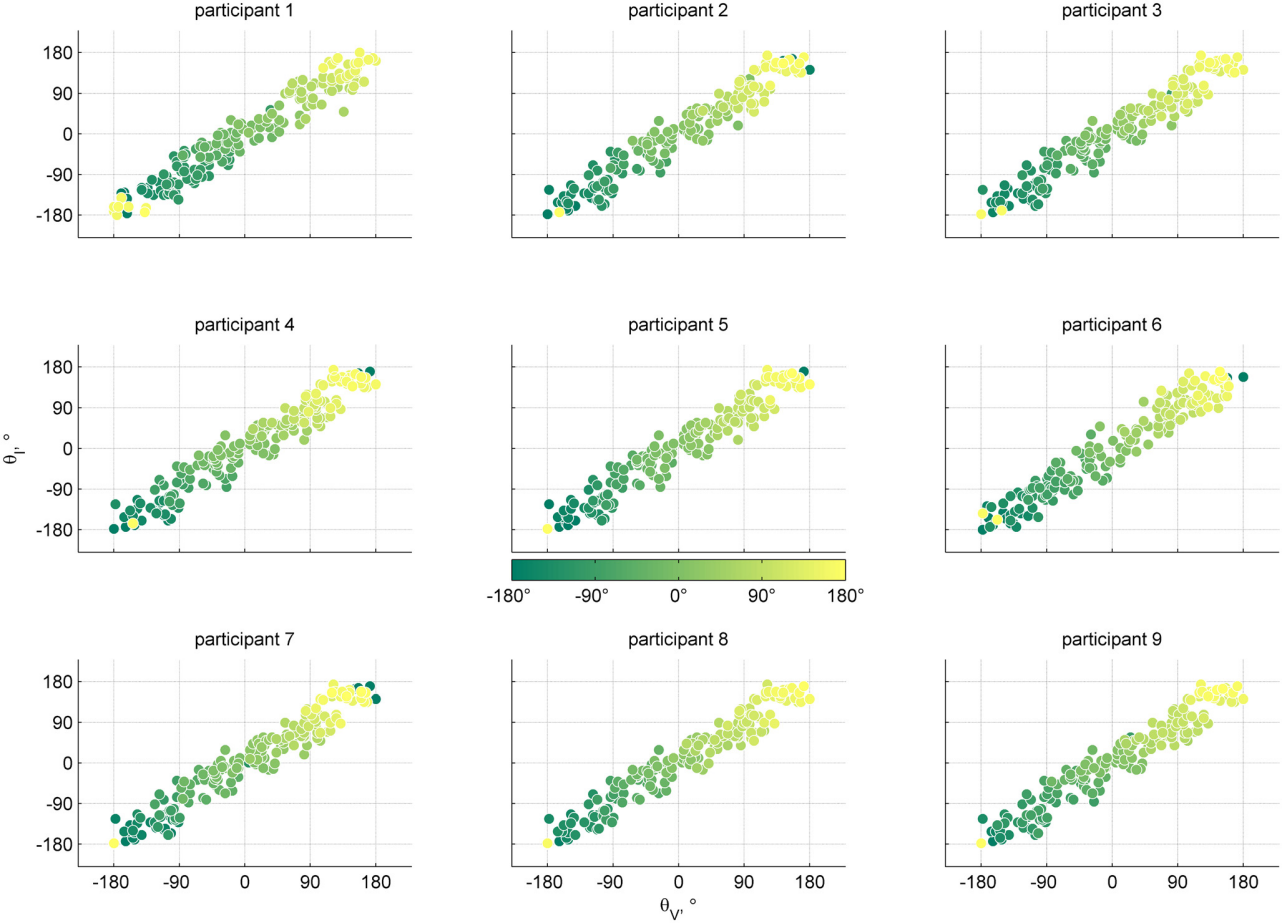
Individual data points for the multisensory condition. The panels show the data for each of the participants separately. The abscissa represents the visual heading angle of multisensory stimuli; the ordinate represents the inertial heading angle. The color of each dot corresponds to the response heading angle. Note that the responses lie on the circle, hence dots colored dark green and bright yellow have a minor angular difference.

For the five participants for whom the BIC scores were in favor of the FF model, the strength of the evidence for preference of this model over the best fitting CI model ranged between a △BIC of 1.58 and 6.54. For the four participants for whom the BIC scores favored the MA model, the △BIC ranged between 0.83 and 5.00. A △BIC ranging between 0–2 is considered weak evidence; 2–6 positive; 6–10 strong; and > 10 is considered decisive evidence [50]. The evidence to discern between the FF and MA models was weak for participants two, five, seven and nine, and positive for participant four. A visual representation of the data of the multisensory conditions is provided in Fig 7.

## Discussion

The present study was designed to investigate how the CNS constructs an estimate of heading for combinations of visual and inertial heading stimuli. Discrepancies between the heading of the visual and inertial stimulus were introduced in order to assess if, and if so how, discrepancies affect the multisensory heading estimation process. Here we compare the present observations on unisensory heading estimation to what has been reported in prior literature and discuss our findings on multisensory heading estimation.

### Unisensory heading estimation

In general, visual heading estimates were found to be biased towards the fore-aft axis, while inertial heading estimates were biased away from the fore-aft axis. The median size of the bias across participants at its maximum was 6.59° in the visual condition, and 2.97° in the inertial condition. These values are in agreement with recent literature [41, 43]. The observed nature of visual bias, i.e., towards the fore-aft axis, is in line with a number of earlier studies [51–54]. In contrast, more recent behavioral studies have found both visual and inertial heading judgments to be biased away from the fore-aft axis [41, 43]. Moreover, the latter findings confirm predictions of population vector decoding (PVD) models. The PVD model makes predictions on perceived heading based on the distribution of preferred directions in populations of neurons. The distribution of preferred directions for otolith afferents has shown a considerable over-representation of lateral headings [2]. Similar findings were reported for preferred direction of populations of neurons in cortical area MSTd [26]. Consequently, the over-representation of the lateral aspect of motion stimuli at the sensory epithelium and in the cortex leads to heading bias away from the fore-aft axis [43].

It has been suggested that the visual bias towards the fore-aft axis that was observed in earlier studies—and the present—may have been caused by presentation of only a limited range of heading stimuli [41]. Although the range of heading stimuli presented has indeed been shown to affect the magnitude of the observed bias [41], it does not reverse the effect; and because a full range of heading stimuli has been presented in the present study while an opposite bias was observed, this explanation is unlikely.

An alternative explanation for the differences in the visual bias patterns observed in the present study compared to others may be found in the nature of the visual stimuli. The studies in which MSTd tuning curves were characterized as well as the recent studies reporting visual bias away from the fore-aft axis all presented visual stimuli with a smaller field of view, a fixation cross, and employed visual stimuli without a ground plane.

Interestingly, fusion of a visual heading estimate with an inertial heading estimate that show opposite bias patterns could lead to a veridical final estimate.

### Multisensory heading estimation

For five out of nine participants in the present study, the best fitting model was the Forced Fusion (FF) model. For three of the four participants for whom the *Model Averaging* Causal Inference (CI) model provided the best fit in an absolute sense, the evidence was not strong enough to clearly distinguish this model from the FF model. Hence, the overall findings are in favor of the FF model. The finding of forced fusion is surprising considering the large discrepancies that were introduced, but it is in line with recent work showing integration in heading estimation for visual and inertial cues with conflicting motion profiles [55], and work showing a similar effect for discrepant visual-inertial information on yaw-rotations [30]. Integration has even been reported for combinations of inertial yaw-rotations and visual roll/pitch motions [31]. For one of our participants, the evidence was in favor of the *Model Averaging* Causal Inference (CI) model. Evidence in favor of a CI model implies that the integration process was affected by discrepancies: the final heading estimate was constructed of an integrated heading estimate on the one hand and an unisensory estimate on the other. It is interesting to note that different people rely on different channels in case a discrepancy was detected by the CNS (according to the model), despite instructions to rely on the inertial motion (Table 2).

In general, our findings imply either that judgments on causality of visual-inertial motion stimuli do not depend on discrepancies in heading angle, or that the detection of discrepancies was impeded by extraneous factors.

In the former case, a possible reason that discrepancies between visual and inertial heading are ignored by the CNS is that these are only encountered in the artificial environments of motion simulators, making an ability to merge causality judgments based on heading discrepancies with actual heading estimates superfluous from an evolutionary standpoint [30]. In this regard, it is interesting to note that in pilot studies conducted for this experiment, one participant remarked feeling that ‘something was off’, without knowing exactly what, when there was an 180° difference in visual and inertial heading angle.

In the latter case, our findings may be explained by other factors: in an earlier study on statistical optimal integration of visual and inertial heading cues, it was found that participants’ responses for multisensory stimuli agreed with the least precise internal signal, instead of with statistically optimal integration of the visual and inertial signal [32]. The results of this study were later explained by the nature of the visual stimuli that were used, which were presented binocularly, but without disparity information. The absence of disparity information may have led to an interpretation of visual stimuli as object-motion, whereas the inertial stimuli were interpreted as self-motion, as it was shown that the incidence of integration is higher for visual stimuli with than without disparity information [28]. Although this explanation fits the observations of a lack of integration, it raises the question why integration was observed in other studies, including the present, as these studies use visual stimuli that are unlikely to elicit vection (i.e. a visually induced sensation of self-motion; [56]), because of the short duration of the motions. Because the motion stimuli used in the study where integration was not observed had a much longer duration (9.3s) than is generally the case, an alternative explanation may be that causal inference is a slow top-down inhibitory process, where discrepancies of a different nature need different times to be detected. Given that people differ in their ability to detect discrepancies, such processing could also explain why causal inference was observed for at least one, and possibly four out of nine participants in the present study.

## Supporting Information

S1 Video Animation of a trial with forward motion. First the cabin is oriented towards the desired heading angle, followed by a 2s break, and subsequently the stimulus’ horizontal linear motion is presented.
(MP4)

S2 Video Animation of a trial with backward motion. First the cabin is oriented towards the desired heading angle, followed by a 2s break, and subsequently the stimulus’ horizontal linear motion is presented.
(MP4)

S1 Table R^2^ unisensory models. Generalized Coefficient of Determination scores, R^2^, for each fitted unisensory model. Scores of zero and one correspond to the worst and best possible fits, respectively.
(PDF)

S2 Table Parameter estimates unisensory models. Parameter estimates for models fitted to data obtained in unisensory conditions. *β*_0_ corresponds to constant bias; *β*_1_ corresponds to heading-dependent bias, where values < 1 indicate bias *towards* and values > 1 *away* from the fore-aft axis; *γ*_0_ is the constant component of the dispersion parameter; and *γ*_1_ corresponds to variability of the dispersion as a function of heading angle. All parameters, except those italicized, are significantly different from zero at the 0.01-level (according to Wald *χ* ^2^-tests).
(PDF)

S1 Dataset MATLAB data file. Structure array containing a matrix field ‘dataset’ with the data gathered in the experiment, and a cell field ‘headers’ specifying matrix column content. The dataset contains data for the nine participants whose results are described in the manuscript (numbered 1–9). Data for two of the excluded participants are included under participant number ‘91’ and ‘92’. Data of the final participant was discarded after the experiment.
(ZIP)
