# Supplementary material for: Forced Fusion in Multisensory Heading Estimation
Source: PLoS One. 2015 May 4;10(5):e0127104. doi: 10.1371/journal.pone.0127104 (PMC4418840; doi:10.1371/journal.pone.0127104)
Supplement: S1 Table — Generalized Coefficient of Determination scores, R2, for each fitted unisensory model. Scores of zero and one correspond to the worst and best possible fits, respectively. (PDF) [file pone.0127104.s003.pdf]

S1 Table

	R^2	
pp	visual	inertial
1	0.780	0.579
2	0.296	0.044
3	0.104	0.042
4	0.604	0.192
5	0.594	0.110
6	0.098	0.170
7	0.320	0.313
8	0.241	0.378
9	0.162	0.439

S1 Table. R^2 **unisensory models.** Generalized Coefficient of Determination scores, R^2, for each fitted unisensory model. Scores of zero and one correspond to the worst and best possible fits, respectively.
