# Supplementary material for: Forced Fusion in Multisensory Heading Estimation
Source: PLoS One. 2015 May 4;10(5):e0127104. doi: 10.1371/journal.pone.0127104 (PMC4418840; doi:10.1371/journal.pone.0127104)
Supplement: S2 Table — Parameter estimates for models fitted to data obtained in unisensory conditions. β 0 corresponds to constant bias; β 1 corresponds to heading-dependent bias, where values < 1 indicate bias towards and values > 1 away from the fore-aft axis; γ 0 is the constant component of the dispersion parameter; and γ 1 corresponds to variability of the dispersion as a function of heading angle. All parameters, except those italicized, are significantly different from zero at the 0.01-level (according to Wald χ 2-tests). (PDF) [file pone.0127104.s004.pdf]

S2 Table

pp	Visual-only				Inertial-only			
	β_0	β_1	γ_0	γ_1	β_0	β_1	γ_0	γ_1
1	-0.31	0.79	97.67	87.63	-0.34	1.04	12.84	*1.22*
2	*0.07*	0.45	5.97	*0.98*	*0.08*	1.09	8.44	*2.04*
3	*0.02*	0.83	18.42	11.16	*0.01*	1.14	17.81	*-2.97*
4	0.08	0.50	23.14	*-1.89*	0.10	0.92	29.67	*13.87*
5	0.04	0.61	71.19	55.79	*0.02*	1.10	53.50	35.77
6	*0.05*	0.92	26.27	16.67	*0.04*	0.90	46.98	36.37
7	0.13	0.72	15.03	*0.27*	0.14	0.71	16.26	*3.35*
8	0.10	1.14	27.63	20.46	0.15	1.13	16.55	*-12.40*
9	0.08	1.12	27.23	*14.52*	0.06	1.41	60.13	50.24

S2 Table. Parameter estimates unisensory models. Parameter estimates for models fitted to data obtained in unisensory conditions. β_0 corresponds to constant bias; β_1 corresponds to heading-dependent bias, where values < 1 indicate bias *towards* and values > 1 *away* from the fore-aft axis; γ_0 is the constant component of the dispersion parameter; and γ_1 corresponds to variability of the dispersion as a function of heading angle. All parameters, except those italicized, are significantly different from zero at the 0.01-level (according to Wald χ^2-tests).
